# The Dynamic Distribution of Porcine Microbiota across Different Ages and Gastrointestinal Tract Segments

**DOI:** 10.1371/journal.pone.0117441

**Published:** 2015-02-17

**Authors:** Wenjing Zhao, Yapeng Wang, Shuyun Liu, Jiaojiao Huang, Zhengxiao Zhai, Chuan He, Jinmei Ding, Jun Wang, Huijuan Wang, Weibing Fan, Jianguo Zhao, He Meng

**Affiliations:** 1 School of Agriculture and Biology, Shanghai Jiao Tong University; Shanghai Key Laboratory of Veterinary Biotechnology, Shanghai, P. R. China; 2 State Key Laboratory of Reproductive Biology, Institute of Zoology, Chinese Academy of Sciences, Chaoyang District, Beijing, P. R. China; 3 Shanghai Personal Biotechnology Limited Company, Shanghai, P. R. China; Chengdu Institute of Biology, CHINA

## Abstract

Metagenome of gut microbes has been implicated in metabolism, immunity, and health maintenance of its host. However, in most of previous studies, the microbiota was sampled from feces instead of gastrointestinal (GI) tract. In this study, we compared the microbial populations from feces at four different developmental stages and contents of four intestinal segments at maturity to examine the dynamic shift of microbiota in pigs and investigated whether adult porcine fecal samples could be used to represent samples of the GI tract. Analysis results revealed that the ratio of *Firmicutes* to *Bacteroidetes* from the feces of the older pigs (2-, 3-, 6- month) were 10 times higher compared to those from piglets (1-month). As the pigs matured, so did it seem that the composition of microbiome became more stable in feces. In adult pigs, there were significant differences in microbial profiles between the contents of the small intestine and large intestine. The dominant genera in the small intestine belonged to aerobe or facultative anaerobe categories, whereas the main genera in the large intestine were all anaerobes. Compared to the GI tract, the composition of microbiome was quite different in feces. The microbial profile in large intestine was more similar to feces than those in the small intestine, with the similarity of 0.75 and 0.38 on average, respectively. Microbial functions, predicted by metagenome profiles, showed the enrichment associated with metabolism pathway and metabolic disease in large intestine and feces while higher abundance of infectious disease, immune function disease, and cancer in small intestine. Fecal microbes also showed enriched function in metabolic pathways compared to microbes from pooled gut contents. Our study extended the understanding of dynamic shift of gut microbes during pig growth and also characterized the profiles of bacterial communities across GI tracts of mature pigs.

## Introduction

The gastrointestinal (GI) microbiome is an enormous and dynamic ecosystem, which not only makes essential products and forms a barrier against the pathogens, but also plays multiple functions in intestinal morphology, immunity development, digestion, and modulating host gene expression [1,2]. Currently, applying the metagenome to investigate human and other mammalian GI microbes has become popular. Aided by fecal microbiota analyses, several studies demonstrated that a series of human diseases, such as obesity, diabetes, and inflammatory bowel disease [3–6], were closely tied to the alterations of gut microbial communities. However, its effectiveness as a model remains in doubt since most experiments were sampled from feces instead of GI tract content. At the same time, the dynamic shifts of intestinal microbiota with age and the GI environment of humans were still unclear. The similarity of size, anatomy and physiology made swine an ideal model for human disease-associated research, particularly for in vivo studies [7,8]. For pigs, microbiota also contribute to development of the gastrointestinal immune system and play casual role in diarrhea [9–11]. In order to justify if the microbiome in feces could be used to represent the composition structure of the microbiome in the intestine, we identified the complete GI microbiota profile of adult pigs and evaluated the similarity to the microbial profile in feces. At the same time, we also systematically investigated the shifts of intestinal microbiota from piglets to adult swine to explore the stability of microbes during maturation.

## Materials and Methods

### Animals and sample collection

Ten Large White pigs, including 7 males and 3 females, were fed a standard swine diet under same husbandry. Piglets were weaned at 30 days of age. After weaning, pigs were fed pre-starter diets for 1 week and fed grower diets thereafter. The ingredients of the diets are provided in S1 Table. Fecal samples were collected at 1, 2, 3, and 6 months of age. At 6 months of age, the pigs were slaughtered, and the contents of four intestinal segments, including jejunum, ileum, cecum, and colon, were collected simultaneously. A total of 80 samples (For each individual, fecal samples were collected from four development stages, and content samples were collected from four intestinal segments) were snap-frozen in liquid nitrogen and stored at -20°C. Protocols used for this experiment were consistent with the Guidelines for the Care and Use of Laboratory Animals established by Beijing Association for Laboratory Animal Science, and approved by the Animal Ethics Committee of Institute of Zoology, Chinese Academy of Sciences.

### Gut microbes 16S rRNA sequencing

Microbial genomic DNA was extracted from fecal and intestinal content samples using the TIANGEN DNA stool mini kit (TIANGEN, cat#DP328) following the manufacturer’s guidelines (http://www.tiangen.com/asset/imsupload/up0921879001368428871.pdf). 11 samples (5 fecal samples and 6 intestinal segmented samples) were discarded because their DNA did not pass our QA criteria. The V4 hypervariable regions of 16S rRNA were amplified by PCR using the barcoded fusion primers referred to our previous study [12]. The PCR condition was as follows: initial denaturation at 94°C for 5 min; 94°C denaturation for 30 sec, 50°C annealing for 30 sec, and 72°C extension for 30 sec, repeated for 25 cycles; final extension at 72°C for 7 min. PCR products were purified using a QIAGEN quick Gel Extraction Kit (QIAGEN, cat# 28706). PCR production from each sample was used to construct sequencing library by using Illumina TruSeq DNA Sample Preparation Kit. For each sample, Barcoded V4 PCR amplicons were sequenced by Illumina Miseq platform. V4 of 16S rRNA amplification and sequencing service were provided by Personal Biotechnology Co., Ltd. (Shanghai, China). Sequence reads were eliminated if containing ambiguous bases, if average phred score was lower than 25, if homopolymer run exceeded 6, if there were mismatches in primers, or if sequence length was shorter than 100bp. Sequences that overlapped the region between R1 and R2, when longer than 10 bp and without any mismatches, were assembled according to their overlap sequence. This step ensured to remove chimera. The sequence reads which could not be assembled were discarded. Barcode and sequencing primers were trimmed from sequence reads. Trimmed and assembled sequences were uploaded to QIIME for further analysis.

### Taxonomy classification and statistical analysis

The trimmed and assembled sequences from each sample were aligned to the RDP 16S rRNA training set 10 using the best hit classification option to classify the taxonomy abundance in QIIME (http://qiime.org/index.html) [13]. Bacterial operation taxonomic units (OTU) were generated using the uclust function in QIIME (http://qiime.org/scripts/pick_otus.html). Ace, chao, shannon index were calculated by mothur. Pie chart and 2d principle component analysis (PCA) results on phylum and genus ranks were generated. Taxonomy abundance at the rank of genus was normalized as follows: (1) The abundance count was transformed by log2. (2) The mean of all transformed values was subtracted from each log transformed measurement, and the difference divided by the standard deviation of all log transformed values for the given sample. Normalized abundance was used to generate heatmap by Cluster3.0 and Java Treeview, and to draw a 3dPCA figure for R (http://rgl.neoscientists.org/about.shtml). Correlation was calculated using Excel. Table 1 lists median of taxonomy abundance of each group.

**Table 1:** Correlation between feces and different GI tract segments for genus abundance.

	ileum(N = 6)	cecum(N = 6)	colon(N = 6)	feces(N = 6)
jejunum(N = 6)	0.905	0.729	0.700	0.390
ileum(N = 6)		0.715	0.690	0.371
cecum(N = 6)			0.914	0.725
colon(N = 6)				0.757

Note: Six samples from each group were used to calculate correlation.

### Microbial function prediction

Microbial function was predicted using PICRUSt [14]. The OTUs were mapped to gg13.5 database at 97% similarity by QIIME’s command “pick_closed_otus”. The OTUs abundance was normalized automatically using 16S rRNA gene copy numbers from known bacterial genomes in Integrated Microbial Genomes (IMG). The predicted genes and their function were aligned to Kyoto Encyclopedia of Genes and Genomes (KEGG) database and the differences among groups were compared through software STAMP (http://kiwi.cs.dal.ca/Software/STAMP) [15]. Two-side Welch’s t-test and Benjamini-Hochberg FDR correction were used in two-group analysis. ANOVA with Tukey-Kramer test and Benjamini-Hochberg correction were chosen for multiple-group analysis.

## Results

### Shifts of microbiota along with body development

**Fig 1:**
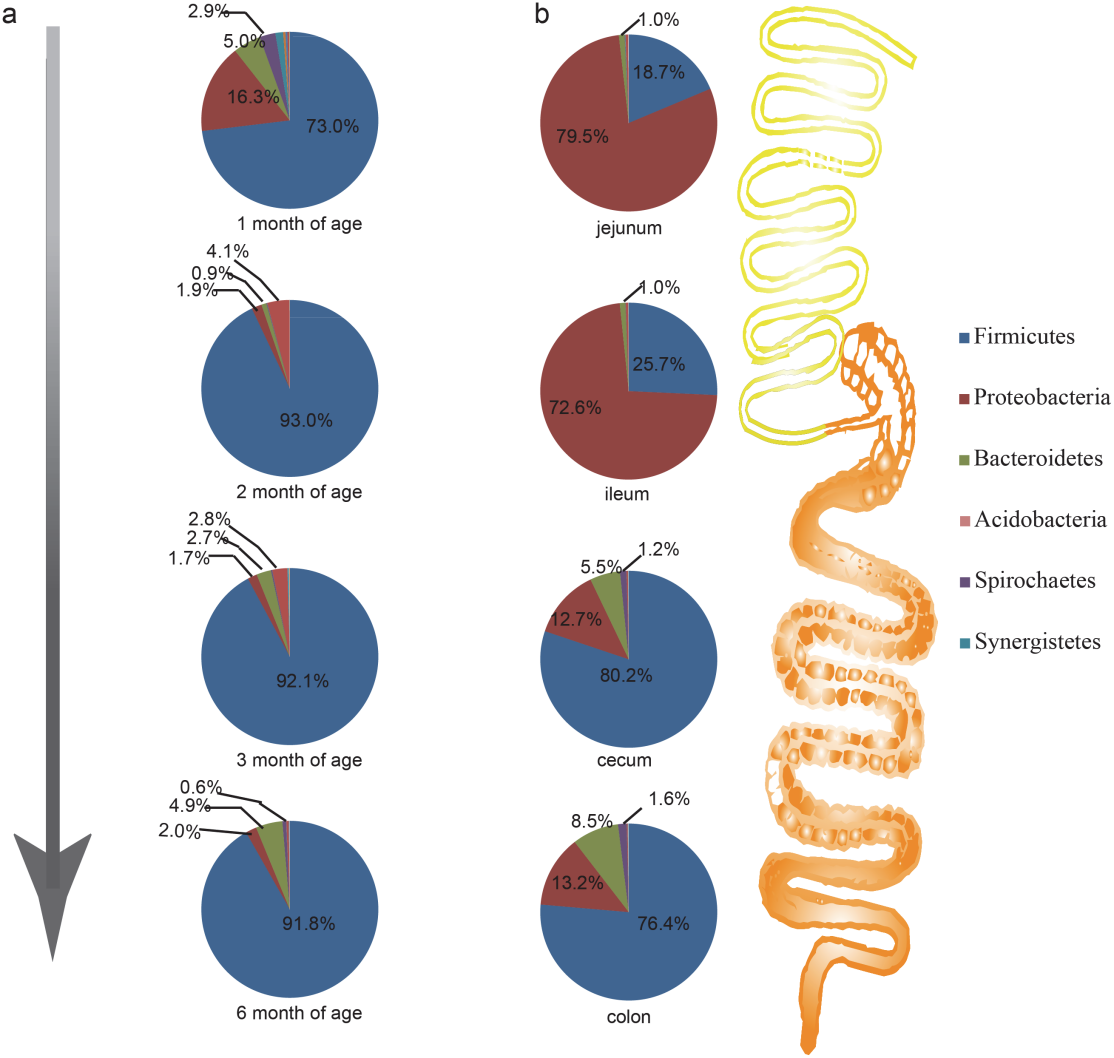
Profiles of gut microbes in GI tracts and feces at the rank of phylum. a, Composition structure of microbiome in feces at of 1, 2, 3, and 6 months of age. b, Profiles of microbes in different GI tract segments.

For the abundance of phylum in feces, *Firmicutes* was the most dominant phylum in all development stages. However, for 1 month old pigs, the abundance of *Firmicutes* (73%) was significantly lower than that of the older pigs (> 90%). Conversely, the *Proteobacteria* was 16.3% in 1 month old pigs and was only about 2% in the older pigs (Fig. 1a). Compared to *Firmicutes* and *Proteobacteria*, *Bacteroidetes* showed a dynamic pattern. The abundance of 5.1% at 1 month of age dropped to 0.9% at 2 months, and then increased to 2.7% and 4.9% at 3 and 6 months of age, respectively. After 1 month, *Actinobacteria* replaced *Spirochaetes* to become the fourth dominant phylum, and it decreased to the fifth at 6 months of age. The ratio of *Firmicutes* to *Bacteroidetes* was 4.48 at 1 month old, increasing more than 10-fold to 50.12 and 53.10 at 2 and 3 months of age, respectively, finally reaching 46.11 at 6 months of age.

On the genus level, ANOVA results revealed that 65 of 172 had significant differences at different ages (*p* < 0.05) (S3 Table). To be more specific in each genus, we further calculated multiple comparisons to show differences between each two ages (S4 Table).

**Fig 2:**
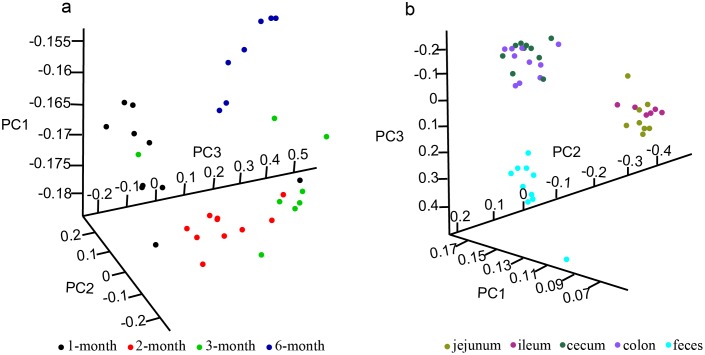
The principal components analysis (PCA) plot of samples from different ages, GI tract, and feces. a, Samples from different ages. b, Samples from feces and different GI tract segments.

PCA results illustrated the difference in distribution of microbiota at the four ages, and 1 month old pigs were distinctly different from other ages (Fig. 2a). On the other hand, the Ace, Chao, Shannon index of 3 and 6 month old pigs were significantly increased compared to 1 and 2 month old pigs, but the Simpson index was slightly lower after 2 months of age (S2 Table). These results suggested that the richness and evenness of microbes in feces were variational along development stage, especially in young pigs (1, 2 month).

### Microbiota profiles in GI tracts of adult pigs

*Proteobacteria* and *Firmicutes* were dominant phyla, making up more than 70% and about 20% of the microbiota in the jejunum and ileum, respectively. Conversely, in the cecum and colon, the *Firmicutes* was most dominant (> 75%), and *Proteobacteria* was about 13% (Fig. 1b).

On the genus level, the PCA analysis clustered samples into the following three categories: small intestine (jejunum and ileum), large intestine (cecum and colon), and feces (Fig. 2b).

**Fig 3:**
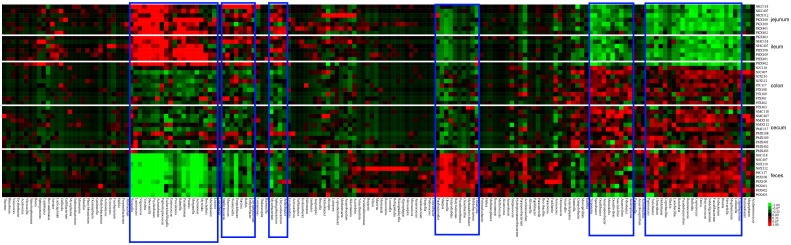
Heatmap of hierarchy cluster results for the abundance of genus in feces and different GI tract segments.

Hierarchy cluster heatmap results confirmed the PCA results and also highlighted the particularly high or low genus in each intestinal segment using a blue frame (Fig. 3). ANOVA results suggested that 97 of 172 total genera showed significantly different abundance in GI tracts (*p* < 0.05) (S3 Table).

**Fig 4:**
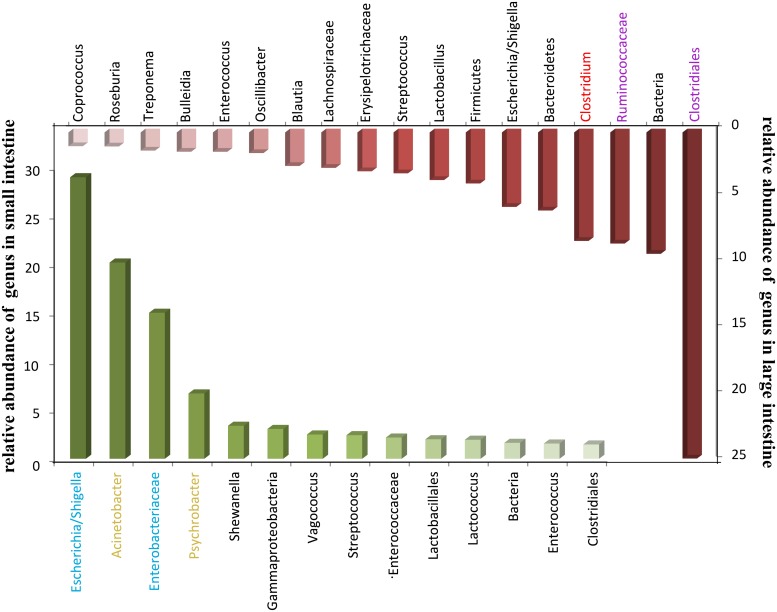
Dominant genus in intestinal tract.

In addition, we compared the enrichment genus of the small intestine with the large intestine. As shown in Fig. 4, *Escherichia* (29.01%), *Acinetobacter* (20.22%), *Enterobacteriaceae Other* (15.04%) and *Psychrobacter* (6.73%) were the top four genera in small intestine, while the dominant genus in large intestine was *Clostridiales* (*Clostridiales Others* 33.89%, *Ruminococcaceae Other* 8.42%, *Clostridium* 8.22%). Blue, yellow, red, or purple genus represented the following types of microbes: facultative anaerobe, aerobe, strict anaerobe, or anaerobe, respectively.

Correlation analysis was conducted to identify the association among the microbiota in different sections of the GI tract. As shown in Table 1, high relevancies were observed between jejunum and ileum (0.905) and colon and cecum (0.914). Moderate relevancies (0.69–0.73) were observed between small intestine (jejunum and ileum) and large intestine (colon and cecum).

**Fig 5:**
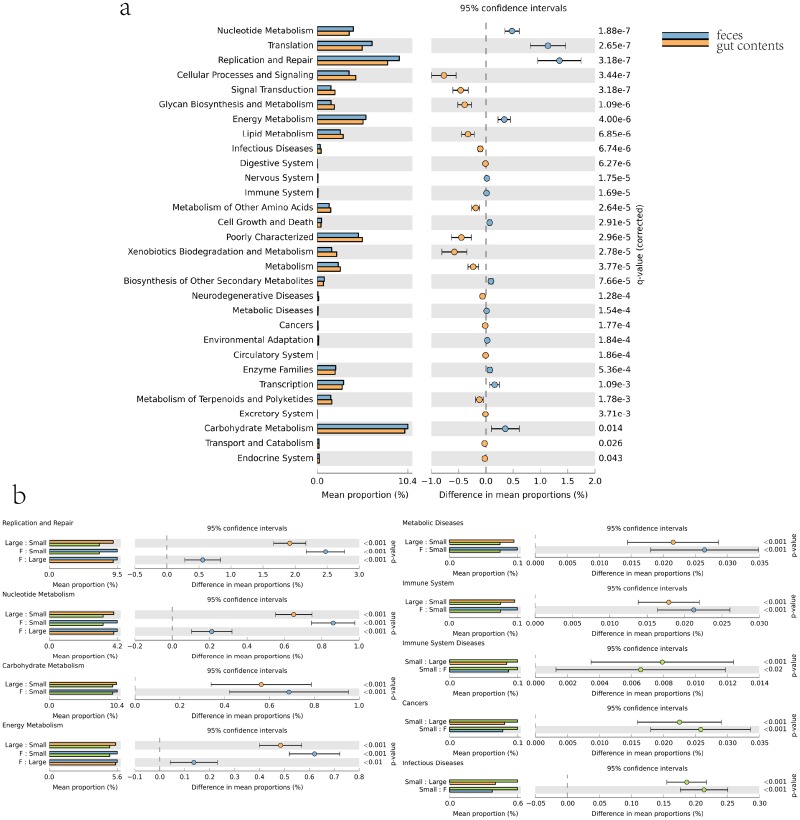
Microbial functional associated with metabolism and disease in intestine and feces. a, Comparison of functional pathway between microbes of feces and gut contents. b, Comparison of microbial functions associated with metabolism and disease among small intestine, large intestine and feces.

In order to investigate the difference in microbiota function between the small and large intestines, we performed functional analysis of microbes using PICRUSt. Microbiota in the small intestine have lower abundance of functions involved in metabolic pathways such as metabolism of carbohydrates, nucleotides, energy, replication and repair, and metabolic disease than those in the large intestine. The small intestine contained fewer immune system-associated microbes, while large intestine contained fewer microbiota associated with immune system disease and cancer (Fig. 5b).

### Relationship of microbiota between feces and intestinal tract

By comparing microbes in feces with the different intestinal segments, we noticed that the profiles of microbes in feces were quite different from those in small intestine. The majority phylum in feces was *Firmicutes* (> 90%), while *Proteobacteria* was the dominant phylum in the small intestine (> 70%) (Fig. 1b). In the large intestine, composition of microbes were more similar to feces both on phylum and genus levels than the small intestine (Fig. 1b, Fig. 2b).

The composition structure of microbiota displayed significant diversity between different GI tract segments and feces. The correlation values ranged from 0.37 to 0.76. The small intestine and feces microbiome communities were quite different (0.37–0.39), while moderately similar between the large intestine and feces (0.73–0.76). Colon and feces showed the highest correlation (0.76) (Table 1).

In terms of the strikingly different genera selected from Fig. 3 and S5 Table, aided by calculating the median and mean of microbial abundance, we discovered the difference between feces and the GI tract (Table 2). The abundance of genera were mostly higher in the GI tract than in feces. For example, in gut contents, *Acinetobacter* was 30 times higher than in feces. Although some genera had lower abundance in feces, they were barely detected in the GI tract, such as *Olsenella* (Table 2).

**Table 2:** Comparisons between feces and different GI tract segments for genus abundance.

Genus	pooled (mean)	pooled (median)	GI tract content (median)	feces (median)	sig. (jejunum)	sig. (ileum)	sig. (cecum)	sig. (colon)
Providencia	4.25	0	0	0	0.000547	0.000221	0.862458	0.938307
Rahnella	13.04545	1	2	0	8.58E-08	6.47E-07	0.224712	0.277698
Brochothrix	25	3	4.5	0	0.006243	0.000101	0.031317	0.003731
Proteus	57.86364	5.5	6.5	0	1.15E-05	1.07E-05	0.036618	0.173058
Myroides	115	7	9.5	0	0.00254	5.53E-06	0.010121	0.00869
Morganella	125.0909	11	16	0	2.14E-08	6.99E-10	0.000148	0.002104
Aeromonas	138.5227	12.5	16.5	0	2.21E-08	1.32E-10	0.000141	0.000197
Pseudomonas	172.3636	20	27	1	1.49E-08	1.87E-07	0.005102	0.011706
Weissella	297.3636	90	147.5	4	1.87E-06	2.15E-06	6.77E-06	0.000792
Peptostreptococcus	408.7273	46.5	68.5	0	3.92E-09	1.41E-10	1.87E-06	6.8E-07
Vagococcus	420.3864	73	110.5	0.5	1.01E-08	3.05E-09	1.15E-05	2.04E-05
Lactococcus	779.0682	186.5	377	0	9.63E-10	9.77E-08	1.86E-08	9.77E-08
Shewanella	801.75	58	83	0	1.46E-12	1.46E-12	9.77E-08	1.06E-07
Yersinia	894.2727	71	136.5	0	1.46E-12	1.93E-11	1.2E-07	2E-07
Enterococcus	1289.659	441.5	809.5	80	0.01025	0.020908	0.005582	0.002058
Psychrobacter	1467.568	152.5	256	3	4.1E-10	2.21E-08	4.97E-05	0.000164
Acinetobacter	4816.75	422.5	671.5	20.5	1.98E-09	1.5E-07	0.000146	7.07E-05
Escherichia/Shigella	7497.136	5249	7712	435	0.000559	0.003426	0.074166	0.116319
Pseudoramibacter	1.068182	0	0	4.5	1.09E-05	6.42E-05	6.97E-07	2.1E-06
Butyricimonas	1.659091	1	0	4	3.51E-05	2.94E-05	7.24E-05	0.000353
Cloacibacillus	2.136364	0	0	7	1.56E-06	1.86E-06	1.06E-07	1.56E-06
Bifidobacterium	3.090909	1	1	5	0.001211	0.001844	0.01025	0.064708
Parabacteroides	4.409091	1	1	9.5	0.021855	9.56E-05	0.000856	8.24E-05
Enterorhabdus	7.795455	1.5	1	26.5	1.96E-06	2.41E-05	0.000221	0.00027
Chlamydia	17.43182	2	1	14.5	0.005194	0.536765	0.036199	0.014128
Olsenella	23.25	1.5	1	63.5	1.44E-09	9.69E-09	3.05E-09	1.17E-09
Sharpea	346.7727	0	0	26.5	0.000206	0.000821	0.000161	0.000353

Comparing predicted microbial function between feces and gut contents, we detected significant enrichment in the metabolism of carbohydrates, nucleotides, energy, and replication and repair in feces.(Fig. 5a). We also investigated microbial functions associated with disease such as metabolic disease, immune system, immune system disease, cancer, and infectious disease. Results suggested that feces contained fewer microbial functions than the small intestine for immune system disease, cancer, and infectious disease but had more functions in metabolic disease and immune function (Fig. 5b).

## Discussion

In mammals, dominant phyla were *Firmicutes*, *Bacteroidetes*, followed by *Fusobacteria*, *Proteobacteria*, *Actinobacteria* [16], but the proportion of each phylum was fluctuant and affected by multiple factors such as animal species. According to our study, adult pigs conformed to this result. Compared to humans, mice, and donkeys, adult pigs had more (>90%) *Firmicutes* in feces [17–20]. Regardless of individual biological variation, in healthy humans, fecal *Bacteroidetes* were much higher than in pigs [21]. Thus, although the intestinal microbial composition is not totally identical to humans, pigs can still be a good candidate for representing humans.

The flexible distribution of intestinal microbes was influnenced by the host beginning at birth [22–25]. For mammals, during development, gut microbiota is influenced by genotype, gender, and reaches stability at maturity. Gut microbiota is also affected by lifestyle on daily timescales [17,26–32]. In pigs, our results demonstrated that the composition of fecal microbes changed during pig development. Microbial profiles from 1 month old pigs were significantly different from those from older pigs (2, 3, and 6 months of age) and had higher relevancy among 2, 3, and 6 month old pigs, which hinted the increasing similarity of gut microbes with each progressive development stage. Porcine gut microbiota became relatively stable at 6 months of age. It had been reported that the *Firmicutes*/*Bacteroidetes* ratio of the human microbiota changed with age [33], and the same trend was observed in our swine study. It had been suggested that fat pigs had more *Firmicutes* but fewer *Bacteroidetes* [3], especially fewer *Bacteroides* which were key at degrading carbohydrates [34]. The increase of *Firmicutes* with pigs’ growth, which we observed in this study, was consistent with the significantly increased fat deposition in older pigs, compared to 1 month old piglets. During development, the diversity and quantity of gut microbiota also increased [33]. In our study, the changing diversity index of Ace, Chao, Shannon, Simpson indicated that the richness and evenness of microbiota at 1 month of age, followed by 2 months, with a disparity when compared to 3 and 6 months of age (S2 Table). In the weaning transition, the diet of the piglets was changed from highly-digestible milk to a less-digestible solid feed (S1 Table). We speculate that besides the age factor, the stress of weaning and shift in food composition might contribute to the significant change in microbiota profiles, as reported [35–37].

Segmented distribution of gut microbes had already been reported in mice, pig, and chicken in previous study [38–40]. In our study, distinct microbial communities between small intestine and large intestine were also found, but the distribution of gut microbes in small intestine did not agree with results in pig on the phylum level. This difference may be caused by porcine species, age, feed, or husbandry. The small intestine is mainly responsible for food digestion and absorption, while the large intestine, especially the cecum, which has high numbers of microorganisms, is related to microbial fermentation [41]. Food passes through the anterior intestine quickly and is retained in the hindgut for several hours [42]. The porcine large intestine contained a larger proportion of *Firmicutes* than small intestine, suggesting that the large intestine, instead of small intestine, might undertake some tasks of fat deposition. Also, the results from microbial function prediction suggested that the small intestine contained less microbiota associated with the metabolic pathway than large intestine (Fig. 5). Because of this, we have reason to believe that microbes in the large intestine undertake more metabolism tasks. The small intestine contained higher microbiota associated with infection, cancer, and the immune system, indicating the pathogen invasion may enrich in this section of the gut. As the closest segment to feces, the large intestine completed the final, lengthy processing of digesta [42]. From our perspective, feces shared the majority of microbes with the large intestine, which suggests that after food digests in the small intestine, gut microbes were selected by the large intestine, based on the digesta, to form feces. *Acinetobacter*, *Escherichia/Shigella*, *Enterococcus*, which were dominant genera in the small intestine, formed the main gap between feces and intestinal microbiota. These results indicate that these genus mostly take part in digestion in the small intestine, and microbes in feces were primarily adapted from the large intestine with a high proportion of functions associated with metabolic pathways and metabolic disease with a lower proportion of functions associated with cancer, and infectious disease. Our results signified that along host GI tracts, distribution of microbes was segmented at the demarcation point of the small and large intestines. In particular, though microbial structure became stable in the host at maturity and feces have a remarkable similarity with the large intestinal microbiome, feces cannot fully represent microbial profiles of GI tracts. This result suggests that when treating bacterial infections caused by a pathogen using fecal transplant, the dynamic shift of gut microbes along the intestinal tract needs to be considered, especially for the shift in small intestine in case of bacterial preference in feces compared to intestinal contents. In conclusion, when using model animals to research the interaction between host and gut microbes associated with health in the future, considering all segments of the intestine could be helpful.

## Supporting Information

S1 Fig The OTU diversity of different groups. a, OTUs of fecal samples from different development ages (1, 2, 3, 6 months of age). b, OTUs of microbes in different GI tract segments. The overlap regions showed the common OTU numbers among groups.
(TIF)

S2 Fig The abundance distribution of different groups in gut microbes of different taxonomic levels. a, Porcine feces grouped by age were enriched with different microbes. b, Porcine GI tract contents grouped by segment was enriched with different microbes. Pie chart showed the proportion separated by different subgroups.
(PDF)

S1 Table Diet ingredients. (DOCX)

S2 Table Diversity index. Indexes of Chao and Ace showed the microbial richness of each sample while indexes of Simpson and Shannon showed the uniformity of structure.
(DOCX)

S3 Table ANOVA results of different ages, feces and different GI tract segments at genus rank. (DOCX)

S4 Table Multiple comparison of feces in different growth ages. (XLSX)

S5 Table Multiple comparison in different intestinal segments and feces. (XLSX)

S6 Table Correlation among different development stages for genus abundance. (DOCX)
