# Supplementary figures and images for: The Dynamic Distribution of Porcine Microbiota across Different Ages and Gastrointestinal Tract Segments
Source: PLoS One. 2015 Feb 17;10(2):e0117441. doi: 10.1371/journal.pone.0117441 (PMC4331431; doi:10.1371/journal.pone.0117441)

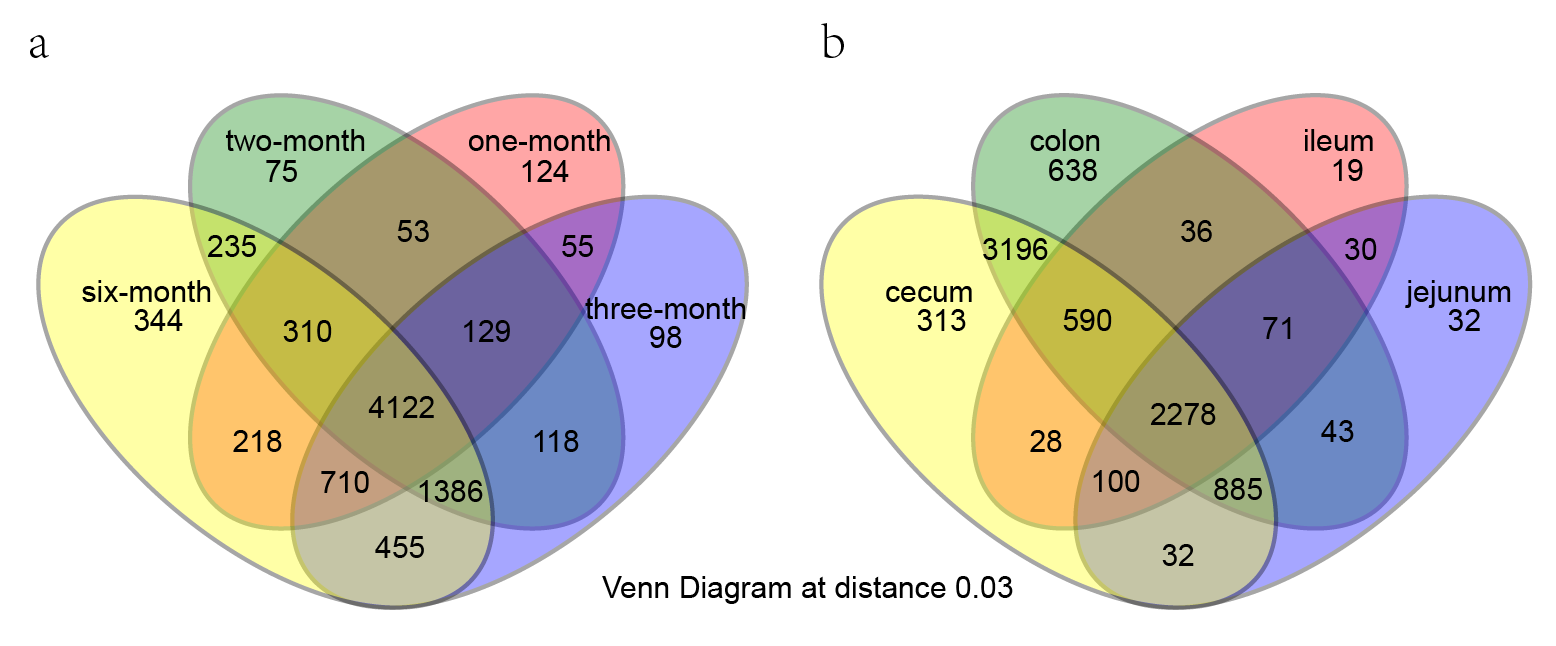

Supplement: S1 Fig — a, OTUs of fecal samples from different development ages (1, 2, 3, 6 months of age). b, OTUs of microbes in different GI tract segments. The overlap regions showed the common OTU numbers among groups. (TIF) [file pone.0117441.s001.tif]

a

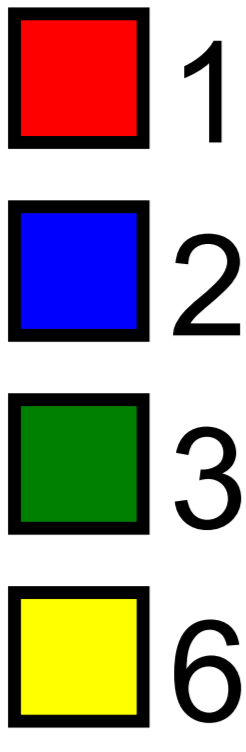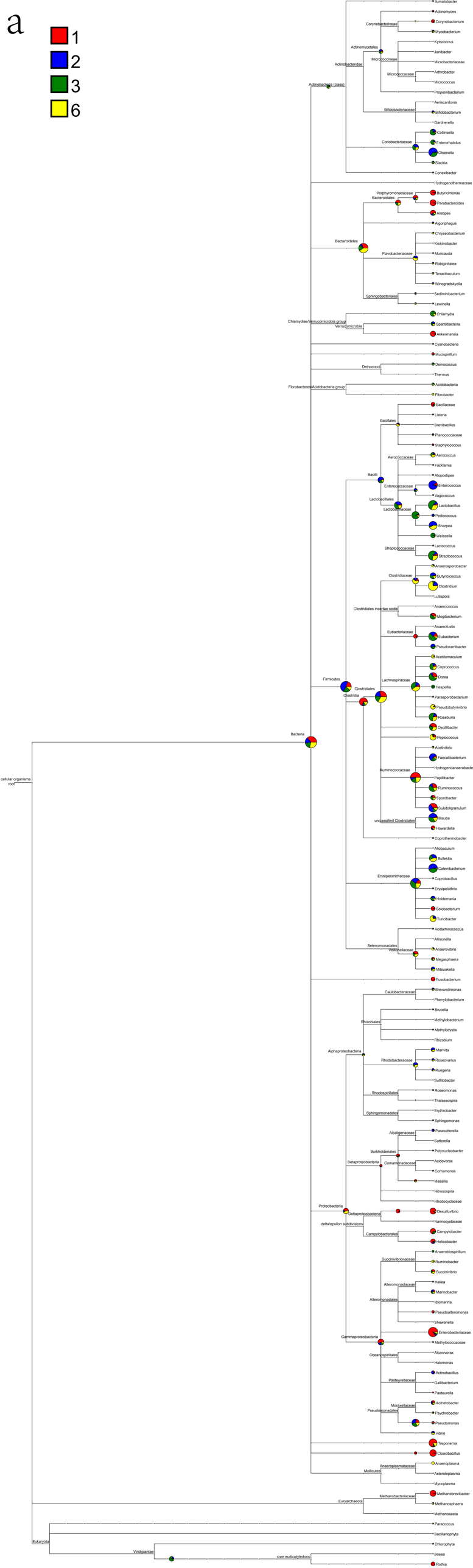

b

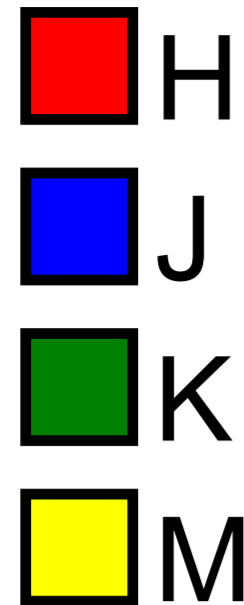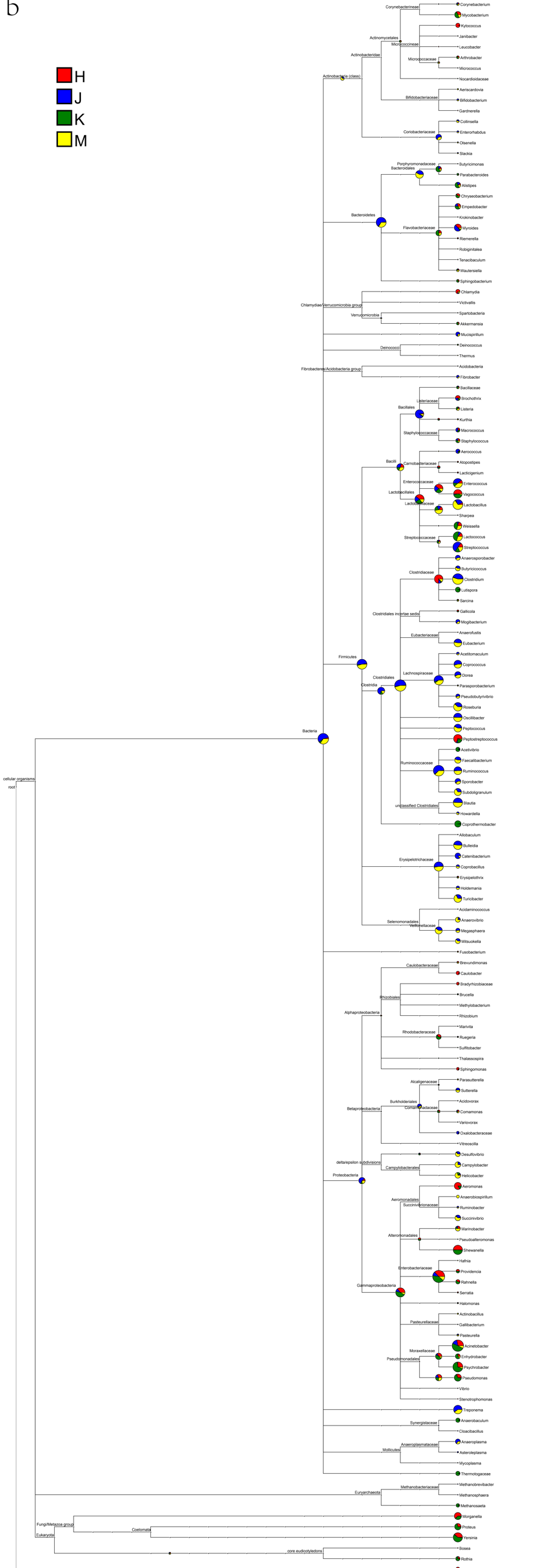

Supplement: S2 Fig — a, Porcine feces grouped by age were enriched with different microbes. b, Porcine GI tract contents grouped by segment was enriched with different microbes. Pie chart showed the proportion separated by different subgroups. (PDF) [file pone.0117441.s002.pdf]
