# Supplementary material for: The Dynamic Distribution of Porcine Microbiota across Different Ages and Gastrointestinal Tract Segments
Source: PLoS One. 2015 Feb 17;10(2):e0117441. doi: 10.1371/journal.pone.0117441 (PMC4331431; doi:10.1371/journal.pone.0117441)
Supplement: S1 Table — (DOCX) [file pone.0117441.s003.docx]

**Table S1. Diet ingredients**

| **Diet type^1^** | **Pre-starter** | **Growing** |
| --- | --- | --- |
| Ingredient (g/kg) |  |  |
| Corn | 402 | 376 |
| Barley |  | 100 |
| Wheat meal | 150 | 75 |
| Soybean meal |  | 95 |
| Swelled soybean meal | 80 |  |
| Fermented soybean meal | 40 |  |
| Peeled soybean meal (46%) | 30 |  |
| Steamed fishmeal (68%) | 30 |  |
| Corn DDGS |  | 125 |
| Rice bran |  | 25 |
| Wheat bran |  | 96 |
| Maize germ meal |  | 50 |
| Ash | 8 | 13 |
| Vegetable oil | 20 | 5 |
| whey powder | 100 |  |
| Molasses | 60 |  |
| Trace elements and vitamin premix^2^ | 80 |  |

^1^Manufacturer: Beijing Sanyuanhefeng farming Lt. C., Beijing, China.

^2^Detailed supplementation not disclosed by the manufacturer.
