# Supplementary material for: The Dynamic Distribution of Porcine Microbiota across Different Ages and Gastrointestinal Tract Segments
Source: PLoS One. 2015 Feb 17;10(2):e0117441. doi: 10.1371/journal.pone.0117441 (PMC4331431; doi:10.1371/journal.pone.0117441)
Supplement: S2 Table — Indexes of Chao and Ace showed the microbial richness of each sample while indexes of Simpson and Shannon showed the uniformity of structure. (DOCX) [file pone.0117441.s004.docx]

**Table S2. Diversity index.** Indexes of Chao and Ace showed the gut microbes’ richness of each sample while indexes of Simpson and Shannon showed the uniformity of structure of GI tract microbes.

| **sample** | **Chao** | **Ace** | **Simpson** | **Shannon** |
| --- | --- | --- | --- | --- |
| N1C118 | 4000.0243 | 4834.5207 | 0.067512 | 4.285963 |
| N1X110 | 4303.1590 | 5439.9463 | 0.041446 | 4.580761 |
| N1X112 | 3547.0792 | 4400.9822 | 0.038417 | 4.410246 |
| P1C117 | 3541.2222 | 4692.4965 | 0.182312 | 3.247888 |
| P1X108 | 4158.0000 | 5435.3384 | 0.074220 | 4.117388 |
| P1X401 | 4283.1022 | 5256.0317 | 0.063585 | 4.276815 |
| P1X403 | 3669.2772 | 4400.0493 | 0.043977 | 4.507301 |
| N2C118 | 4335.1725 | 4465.6893 | 0.050202 | 4.416947 |
| N2C407 | 3606.9375 | 4987.5275 | 0.106799 | 3.588222 |
| N2X110 | 3497.7230 | 4203.7412 | 0.039010 | 4.235017 |
| N2X112 | 3854.9322 | 4574.1569 | 0.138858 | 3.796341 |
| P2X109 | 3027.1608 | 3944.5526 | 0.080819 | 3.502718 |
| P2X401 | 3977.7063 | 4820.7825 | 0.084245 | 4.034188 |
| P2X402 | 3756.1232 | 4353.5038 | 0.069412 | 4.306636 |
| P2X403 | 5616.3549 | 7163.1640 | 0.025247 | 5.256093 |
| N3C118 | 4695.3399 | 5782.5089 | 0.074264 | 4.289657 |
| N3C407 | 4696.6038 | 5926.1864 | 0.027085 | 5.003141 |
| N3X110 | 5095.5985 | 6095.3272 | 0.029099 | 5.031643 |
| N3X112 | 5080.2053 | 5143.8869 | 0.037189 | 4.740368 |
| P3C117 | 4548.0745 | 5400.1468 | 0.033751 | 4.661576 |
| P3X108 | 4944.4307 | 6199.9260 | 0.020300 | 5.324662 |
| P3X109 | 4009.0845 | 5006.6990 | 0.039808 | 4.608872 |
| P3X401 | 4113.9240 | 4095.3789 | 0.060478 | 4.315111 |
| P3X402 | 5075.3321 | 6280.086 | 0.040801 | 4.836711 |
| P3X403 | 3826.7373 | 4654.6996 | 0.060624 | 4.207686 |
| N6C118 | 5776.4246 | 7186.2293 | 0.035232 | 5.055006 |
| N6C407 | 6114.3865 | 7633.6737 | 0.024026 | 5.273260 |
| N6X110 | 5867.8000 | 7158.8267 | 0.020131 | 5.304090 |
| N6X112 | 6265.0740 | 6359.4763 | 0.027156 | 5.328836 |
| P6C117 | 5813.2489 | 7250.3504 | 0.035793 | 4.977577 |
| P6X108 | 6674.2617 | 6940.9503 | 0.014394 | 5.668873 |
| P6X109 | 5181.9182 | 6311.2004 | 0.021227 | 5.196272 |
| P6X401 | 4547.2654 | 5697.6869 | 0.095515 | 4.039713 |
| P6X402 | 5375.4961 | 6667.0497 | 0.035503 | 5.049479 |
| P6X403 | 3447.439 | 4526.752 | 0.108476 | 3.926106 |
| NKC118 | 2319.2231 | 2969.9495 | 0.108821 | 3.182411 |
| NKC407 | 1720.0000 | 2222.4813 | 0.542985 | 1.845152 |
| NKX112 | 2489.9381 | 2950.599 | 0.078556 | 3.633269 |
| PKX108 | 2400.9597 | 3058.1382 | 0.071197 | 3.643678 |
| PKX109 | 2275.8610 | 2786.5543 | 0.138401 | 2.964517 |
| PKX401 | 2477.4054 | 2457.9979 | 0.100533 | 3.295175 |
| PKX402 | 2966.7906 | 3777.8325 | 0.057193 | 3.791446 |
| PKX403 | 2025.2772 | 2657.0177 | 0.073429 | 3.529248 |
| NHC118 | 2564.8508 | 2601.1765 | 0.342300 | 2.651963 |
| NHC407 | 1910.0821 | 2599.6654 | 0.365166 | 2.548618 |
| PHX108 | 2294.8268 | 2946.8541 | 0.053804 | 3.893150 |
| PHX109 | 2575.3333 | 3247.7673 | 0.053177 | 3.832325 |
| PHX401 | 2292.306 | 3022.0331 | 0.126874 | 3.316221 |
| PHX402 | 2507.25 | 3083.8944 | 0.066887 | 3.542607 |
| NMC118 | 5194.6338 | 5201.2033 | 0.020266 | 5.157524 |
| NMC407 | 4492.9219 | 5852.6772 | 0.025405 | 5.16447 |
| NMX110 | 5834.4091 | 7190.2146 | 0.019291 | 5.181773 |
| NMX112 | 4382.3631 | 5736.5619 | 0.078586 | 4.049196 |
| PMC117 | 5526.9262 | 6869.7313 | 0.016590 | 5.266215 |
| PMX108 | 4873.4520 | 6246.7585 | 0.043000 | 4.548837 |
| PMX109 | 4928.0289 | 6437.4679 | 0.068490 | 4.196415 |
| PMX401 | 5255.2145 | 5390.3767 | 0.024795 | 4.968665 |
| PMX402 | 5185.8055 | 6446.6985 | 0.026965 | 4.824851 |
| PMX403 | 4650.2590 | 6042.9843 | 0.113168 | 4.251766 |
| NJC118 | 5181.1817 | 6488.174 | 0.053707 | 4.828114 |
| NJC407 | 4908.2554 | 6254.1294 | 0.017957 | 5.347541 |
| NJX110 | 5809.2727 | 7469.5577 | 0.022065 | 5.239886 |
| NJX112 | 5635.1630 | 6991.3993 | 0.023199 | 5.229169 |
| PJC117 | 5962.5091 | 7183.5242 | 0.020159 | 5.293559 |
| PJX108 | 4955.6250 | 6455.8028 | 0.030448 | 5.023201 |
| PJX109 | 5793.9407 | 5983.3236 | 0.019366 | 5.414097 |
| PJX401 | 5167.9051 | 6280.3603 | 0.082056 | 4.166292 |
| PJX402 | 5634.7064 | 6896.9674 | 0.025828 | 4.971695 |
| PJX403 | 5136.4622 | 6357.5676 | 0.023087 | 5.230588 |
| p-value(1,2,3,6) | 7.53E-05 | 0.000221 | 0.086293 | 0.002544 |
| p-value(K,H,M,J,6) | 5.27E-17 | 2.78E-18 | 0.00725 | 1.79E-10 |
