# Supplementary material for: The Dynamic Distribution of Porcine Microbiota across Different Ages and Gastrointestinal Tract Segments
Source: PLoS One. 2015 Feb 17;10(2):e0117441. doi: 10.1371/journal.pone.0117441 (PMC4331431; doi:10.1371/journal.pone.0117441)
Supplement: S3 Table — (DOCX) [file pone.0117441.s005.docx]

**Table S3. One-way ANOVA for genus abundance** (******p<0.05*)

| **Genus** | **GI tracts segments** |  | **Age** |  |
| --- | --- | --- | --- | --- |
| Acetitomaculum | 3.35E-05 | * | 0.005656 | * |
| Acetivibrio | 0.113548 |  | 0.553973 |  |
| Acidaminococcus | 0.594502 |  | 0.879992 |  |
| Acidovorax | 0.392355 |  | 0.288241 |  |
| Acinetobacter | 0.306028 |  | 2.51E-09 | * |
| Actinobacillus | 0.245576 |  | 0.112188 |  |
| Actinomyces | 0.036842 | * | 0.751072 |  |
| Aeriscardovia | 0.594502 |  | 0.426369 |  |
| Aerococcus | 0.950221 |  | 0.006959 | * |
| Aeromonas | 0.54254 |  | 1.53E-10 | * |
| Akkermansia | 0.02829 | * | 0.18397 |  |
| Alcanivorax | 0.245576 |  | 0.30303 |  |
| Algoriphagus | 0.245576 |  | 0.040292 | * |
| Alistipes | 0.110573 |  | 0.319881 |  |
| Allisonella | 0.594502 |  | 0.235279 |  |
| Allobaculum | 0.057023 |  | 0.248154 |  |
| Anaerobaculum | 0.985969 |  | 0.235279 |  |
| Anaerobiospirillum | 0.049965 | * | 0.20739 |  |
| Anaerococcus | 0.769519 |  | 0.671493 |  |
| Anaerofustis | 0.375543 |  | 0.223533 |  |
| Anaeroplasma | 0.069119 |  | 0.112188 |  |
| Anaerosporobacter | 0.000474 | * | 3.04E-06 | * |
| Anaerovibrio | 0.021842 | * | 0.006718 | * |
| Arthrobacter | 0.594502 |  | 0.231973 |  |
| Asteroleplasma | 0.161746 |  | 0.496896 |  |
| Atopostipes | 0.310236 |  | 0.098652 |  |
| Bifidobacterium | 0.316716 |  | 0.001891 | * |
| Blautia | 0.006046 | * | 4.76E-10 | * |
| Bosea | 0.328577 |  | 0.031635 | * |
| Brevibacillus | 0.255608 |  | 0.974046 |  |
| Brevundimonas | 0.101573 |  | 0.012731 | * |
| Brochothrix | 0.594502 |  | 0.000528 | * |
| Brucella | 0.208518 |  | 0.176018 |  |
| Bulleidia | 0.000933 | * | 7.34E-08 | * |
| Butyricicoccus | 0.123116 |  | 1.70E-05 | * |
| Butyricimonas | 2.41E-06 | * | 1.66E-05 | * |
| Campylobacter | 0.028155 | * | 0.001305 | * |
| Catenibacterium | 5.09E-06 | * | 6.47E-07 | * |
| Caulobacter | 0.848764 |  | 0.329342 |  |
| Chlamydia | 0.028155 | * | 0.012303 | * |
| Chryseobacterium | 0.27213 |  | 0.008052 | * |
| Cloacibacillus | 2.34E-06 | * | 6.77E-08 | * |
| Clostridium | 0.000182 | * | 0.00016 | * |
| Collinsella | 0.004164 | * | 2.24E-07 | * |
| Comamonas | 0.245576 |  | 0.114199 |  |
| Conexibacter | 0.745555 |  | 0.039352 | * |
| Coprobacillus | 0.005097 | * | 0.002353 | * |
| Coprococcus | 0.007835 | * | 6.70E-08 | * |
| Coprothermobacter | 0.594502 |  | 0.108689 |  |
| Corynebacterium | 0.032169 | * | 0.776442 |  |
| Deinococcus | 0.765861 |  | 0.544484 |  |
| Desulfovibrio | 0.000783 | * | 0.017878 | * |
| Dorea | 0.000939 | * | 5.27E-08 | * |
| Empedobacter | 0.594502 |  | 0.000433 | * |
| Enhydrobacter | 0.848764 |  | 6.30E-05 | * |
| Enterococcus | 0.000212 | * | 0.005384 | * |
| Enterorhabdus | 2.63E-05 | * | 4.01E-06 | * |
| Erysipelothrix | 0.630199 |  | 0.769671 |  |
| Erythrobacter | 0.006612 | * | 0.211724 |  |
| Escherichia/Shigella | 3.24E-05 | * | 0.001676 | * |
| Eubacterium | 0.014633 | * | 3.25E-10 | * |
| Facklamia | 0.186641 |  | 0.000902 | * |
| Faecalibacterium | 0.288654 |  | 8.49E-07 | * |
| Fibrobacter | 0.021842 | * | 0.086596 |  |
| Fusobacterium | 0.000847 | * | 0.776442 |  |
| Gallibacterium | 0.717102 |  | 0.093504 |  |
| Gallicola | 0.594502 |  | 0.014813 | * |
| Gardnerella | 0.285325 |  | 0.010751 | * |
| Hafnia | 0.594502 |  | 0.136608 |  |
| Haliea | 0.647322 |  | 0.000767 | * |
| Halomonas | 0.164094 |  | 0.283076 |  |
| Helicobacter | 0.032169 | * | 0.102232 |  |
| Hespellia | 0.0324 | * | 0.01371 | * |
| Holdemania | 0.032924 | * | 0.000831 | * |
| Howardella | 0.0324 | * | 0.00035 | * |
| Hydrogenoanaerobacterium | 0.11314 |  | 0.283076 |  |
| Idiomarina | 0.653892 |  | 0.752793 |  |
| Janibacter | 0.708942 |  | 0.117452 |  |
| Krokinobacter | 0.057025 |  | 0.046814 | * |
| Kurthia | 0.950221 |  | 0.001313 | * |
| Kytococcus | 0.557332 |  | 0.012731 | * |
| Lacticigenium | 0.594502 |  | 0.010751 | * |
| Lactobacillus | 0.00095 | * | 0.000735 | * |
| Lactococcus | 0.848764 |  | 4.76E-10 | * |
| Leucobacter | 0.594502 |  | 0.720359 |  |
| Lewinella | 0.697062 |  | 0.047033 | * |
| Listeria | 0.594502 |  | 0.058863 |  |
| Lutispora | 0.354633 |  | 0.254459 |  |
| Macrococcus | 0.435767 |  | 0.003994 | * |
| Marinobacter | 0.898396 |  | 0.054414 |  |
| Marivita | 0.922138 |  | 0.087848 |  |
| Massilia | 0.594502 |  | 0.405056 |  |
| Megasphaera | 0.594502 |  | 0.235279 |  |
| Methanobrevibacter | 2.49E-09 | * | 0.039352 | * |
| Methanosaeta | 0.306028 |  | 0.200529 |  |
| Methanosphaera | 0.583874 |  | 0.14911 |  |
| Methylobacterium | 0.764386 |  | 0.114199 |  |
| Methylocystis | 0.848764 |  | 0.050453 |  |
| Micrococcus | 0.907121 |  | 0.759723 |  |
| Mitsuokella | 0.186641 |  | 0.011637 | * |
| Mogibacterium | 0.028155 | * | 7.34E-08 | * |
| Morganella | 0.594502 |  | 3.26E-10 | * |
| Mucispirillum | 0.030573 | * | 0.000216 | * |
| Muricauda | 0.437481 |  | 0.037559 | * |
| Mycobacterium | 0.925063 |  | 0.010714 | * |
| Mycoplasma | 0.148566 |  | 0.776442 |  |
| Myroides | 0.98158 |  | 4.81E-05 | * |
| Nitrosospira | 0.750648 |  | 0.283076 |  |
| Oceanisphaera | 0.717102 |  | 0.472672 |  |
| Olsenella | 0.000182 | * | 1.53E-10 | * |
| Oscillibacter | 0.008977 | * | 4.83E-07 | * |
| Papillibacter | 0.159616 |  | 0.932041 |  |
| Parabacteroides | 6.13E-05 | * | 8.01E-05 | * |
| Paracoccus | 0.717102 |  | 0.355694 |  |
| Parasporobacterium | 0.316716 |  | 0.384032 |  |
| Parasutterella | 0.204016 |  | 0.833559 |  |
| Pasteurella | 0.011814 | * | 0.088904 |  |
| Pediococcus | 0.037402 | * | 0.132049 |  |
| Peptococcus | 0.006845 | * | 0.000301 | * |
| Peptostreptococcus | 0.594502 |  | 1.53E-10 | * |
| Phenylobacterium | 0.162318 |  | 0.496848 |  |
| Polynucleobacter | 0.452636 |  | 0.005453 | * |
| Propionibacterium | 0.145214 |  | 0.496458 |  |
| Proteus | 0.594502 |  | 3.46E-06 | * |
| Providencia | 0.594502 |  | 2.41E-05 | * |
| Pseudoalteromonas | 0.456528 |  | 0.000735 | * |
| Pseudobutyrivibrio | 8.46E-05 | * | 3.13E-05 | * |
| Pseudomonas | 0.110573 |  | 5.27E-09 | * |
| Pseudoramibacter | 0.186641 |  | 6.47E-07 | * |
| Psychrobacter | 0.015431 | * | 3.26E-10 | * |
| Rahnella | 0.594502 |  | 5.89E-09 | * |
| Rhizobium | 0.910546 |  | 0.377595 |  |
| Riemerella | 0.854209 |  | 0.319881 |  |
| Robiginitalea | 0.594502 |  | 0.054054 |  |
| Roseburia | 0.088355 |  | 1.13E-05 | * |
| Roseomonas | 0.594502 |  | 0.107633 |  |
| Roseovarius | 0.594502 |  | 0.086596 |  |
| Rothia | 0.014633 | * | 0.114199 |  |
| Ruegeria | 0.284546 |  | 0.187294 |  |
| Ruminobacter | 0.049965 | * | 0.017878 | * |
| Ruminococcus | 0.702828 |  | 5.27E-08 | * |
| Sarcina | 0.986201 |  | 0.265717 |  |
| Sediminibacterium | 0.765861 |  | 0.405056 |  |
| Serratia | 0.594502 |  | 0.000338 | * |
| Sharpea | 0.028155 | * | 0.000124 | * |
| Shewanella | 0.186641 |  | 3.13E-13 | * |
| Slackia | 0.028155 | * | 0.002745 | * |
| Solobacterium | 0.068349 |  | 0.025954 | * |
| Sphingobacterium | 0.416355 |  | 0.001164 | * |
| Sphingomonas | 0.67003 |  | 0.108689 |  |
| Sporobacter | 0.148566 |  | 2.41E-05 | * |
| Staphylococcus | 0.709463 |  | 0.116884 |  |
| Stenotrophomonas | 0.204252 |  | 0.047033 | * |
| Streptococcus | 0.018312 | * | 0.098652 |  |
| Subdoligranulum | 0.088355 |  | 4.50E-05 | * |
| Succinivibrio | 0.146187 |  | 0.006715 | * |
| Sulfitobacter | 0.0324 | * | 0.149324 |  |
| Sutterella | 0.03799 | * | 5.35E-05 | * |
| Tenacibaculum | 0.902502 |  | 0.150379 |  |
| Thalassospira | 0.799325 |  | 0.89832 |  |
| Thermus | 0.240236 |  | 0.776442 |  |
| Treponema | 0.038785 | * | 3.96E-09 | * |
| Turicibacter | 0.00396 | * | 0.00715 | * |
| Vagococcus | 0.294551 |  | 1.15E-09 | * |
| Variovorax | 0.717102 |  | 0.30558 |  |
| Vibrio | 0.594502 |  | 0.176018 |  |
| Victivallis | 0.245576 |  | 0.102232 |  |
| Vitreoscilla | 0.594502 |  | 0.111096 |  |
| Wautersiella | 0.54254 |  | 0.285031 |  |
| Weissella | 0.042308 | * | 6.47E-07 | * |
| Winogradskyella | 0.848764 |  | 0.106824 |  |
| Yersinia | 0.432664 |  | 8.01E-13 | * |
