# Supplementary material for: The Dynamic Distribution of Porcine Microbiota across Different Ages and Gastrointestinal Tract Segments
Source: PLoS One. 2015 Feb 17;10(2):e0117441. doi: 10.1371/journal.pone.0117441 (PMC4331431; doi:10.1371/journal.pone.0117441)
Supplement: S6 Table — (DOCX) [file pone.0117441.s008.docx]

**Table S6. Correlation among different development stages for genus abundance**

|  | 2-month  (N=5) | 3-month (N=5) | 6-month  (N=5) |
| --- | --- | --- | --- |
| 1 month (N=5) | 0.750 | 0.754 | 0.741 |
| 2 month (N=5) |  | 0.836 | 0.794 |
| 3 month (N=5) |  |  | 0.831 |
